# Natalizumab continuation versus switching to ocrelizumab after PML risk stratification in RRMS patients: a natural experiment

**DOI:** 10.1007/s00415-023-11645-x

**Published:** 2023-03-13

**Authors:** Albert Muñoz-Vendrell, Pablo Arroyo-Pereiro, Isabel León, Laura Bau, Elisabet Matas, Antonio Martínez-Yélamos, Sergio Martínez-Yélamos, Lucía Romero-Pinel

**Affiliations:** Neurology Department, Multiple Sclerosis Unit, Hospital Universitari de Bellvitge-IDIBELL, Universitat de Barcelona, L’Hospitalet de Llobregat, Carrer de la Feixa Llarga S/N, 08907 Barcelona, Spain

**Keywords:** JC virus, Natural experiment, Pseudo-randomization, Natalizumab, Ocrelizumab, Switching

## Abstract

**Background:**

Natalizumab (NTZ) and ocrelizumab (OCR) can be used for the treatment of relapsing–remitting multiple sclerosis (RRMS). In patients treated with NTZ, screening for JC virus (JCV) is mandatory, and a positive serology usually requires a change in treatment after 2 years. In this study, JCV serology was used as a natural experiment to pseudo-randomize patients into NTZ continuation or OCR.

**Methods:**

An observational analysis of patients who had received NTZ for at least 2 years and were either changed to OCR or maintained on NTZ, depending on JCV serology status, was performed. A stratification moment (STRm) was established when patients were pseudo-randomized to either arm (NTZ continuation if JCV negativity, or change to OCR if JCV positivity). Primary endpoints include time to first relapse and presence of relapses after STRm and OCR initiation. Secondary endpoints include clinical and radiological outcomes after 1 year.

**Results:**

Of the 67 patients included, 40 continued on NTZ (60%) and 27 were changed to OCR (40%). Baseline characteristics were similar. Time to first relapse was not significantly different. Ten patients in the JCV + OCR arm presented a relapse after STRm (37%), four during the washout period, and 13 patients in the JCV-NTZ arm (32.5%, *p* = 0.701). No differences in secondary endpoints were detected in the first year after STRm.

**Conclusions:**

The JCV status can be used as a natural experiment to compare treatment arms with a low selection bias. In our study, switching to OCR versus NTZ continuation led to similar disease activity outcomes.

## Introduction

Since its approval for the treatment of relapsing–remitting multiple sclerosis (RRMS), natalizumab (NTZ) has shown significant benefits in reducing disease activity [1–4]. However, cases of human polyomavirus JC virus (JCV) reactivation causing progressive multifocal leukoencephalopathy (PML) have generated a widespread concern [5] and led to the implementation of serial JCV serology tests in patients undergoing NTZ treatment to stratify their risk [6, 7].

Since 2019, ocrelizumab (OCR) has been approved as a highly effective treatment for RRMS [8]. In contrast to NTZ, only anecdotal cases of PML have occurred with OCR [9], thus serving as a good alternative to NTZ when JCV serologies are positive [10–12]. However, NTZ cessation is associated with an increased risk of relapse [13, 14], and although there is no evidence of multiple sclerosis (MS) activity after changing to OCR, this therapeutic strategy may pose a risk of MS reactivation. In fact, previous studies have shown the reactivation of MS when changing from NTZ to fingolimod [15], especially if the washout period was prolonged for more than 2 months [16].

In both social and health research, a study design based on a natural experiment has been proposed [17]. An exogenous factor, such as a regional law or a natural phenomenon, allows the creation of groups with low selection bias, thus allowing its comparison with similar efficiency to a randomized treatment and control design.

This study aimed to compare therapeutic strategies for switching to OCR or continuing on NTZ after at least two years of NTZ treatment based on JCV serology, which works as a natural pseudo-randomization factor. We compared the disease activity between the two groups during follow-up.

## Methods

### Study design and participants

This observational study included patients admitted to the MS unit of a tertiary referral hospital (Bellvitge University Hospital, Barcelona, Spain). Patients diagnosed with relapsing–remitting multiple sclerosis (RRMS) according to the McDonald criteria [18, 19] and who had received NTZ for at least 2 years between January 2018 and March 2021 were evaluated for the study. Patients who were either changed to OCR or maintained on NTZ and with at least one year of follow-up after PML risk stratification were selected. Patients were excluded if NTZ was discontinued for any reason other than JCV positivity or if it was changed to another therapy different from OCR.

The PML risk stratification moment (STRm) was established for the first time when JC virus antibody levels were assessed between January 2018 and March 2021. Patients were classified according to their treatment after STRm into continued NTZ (in case of JCV negativity) or changed to OCR (in case of JCV positivity) after a minimum of two months of planned washout. The treatment depended on the JC virus status at the discretion of the attending physicians and under expert committee approval. JCV positivity was considered when a minimum titer of 0.4 was obtained.

The patients received intravenous NTZ every 4 weeks and intravenous OCR every 6 months. During washout periods, all patients who changed NTZ received monthly doses of one gram of intravenous methylprednisolone.

### Outcome measures

The European Database for Multiple Sclerosis (EDMUS) software was used for the prospective collection of clinical and imaging data [20]. Clinical data were obtained by a qualified neurologist from 6-monthly follow-up visits to our specialized MS unit plus additional visits in cases of suspected relapse. A relapse was considered if the patient presented consistent new symptoms or signs lasting more than 24 h in the absence of any intercurrence, without need for EDSS increase or MRI abnormalities. Magnetic resonance imaging (MRI) scans were performed annually per protocol for all patients, plus MRIs within three months before OCR first dose and at three months after treatment initiation. All the MRI scans were evaluated by a qualified neuroradiologist.

The variables studied included the number of relapses during the two years before STRm and the number of annual relapses after STRm, time to first relapse after STRm and after OCR initiation, the Expanded Disability Status Scale (EDSS) score obtained every 6 months, T2 new hyperintense lesions, and gadolinium-enhancing lesions on the basal MRI prior to STRm and in annual controls after that, plus an additional MRI obtained 3 months before and after OCR initiation. The proportion of patients with no evidence of disease activity (NEDA-3) after 1 year and 2 years of follow-up were evaluated. The NEDA-3 definition used included the absence of relapse, no evidence of increased EDSS (defined as ≥ 1.5-point EDSS increase if EDSS = 0, ≥ 1-point EDSS increase if EDSS 1–5.5, or ≥ 0.5-point EDSS increase if EDSS ≥ 6.0), and no new T2-hyperintense lesions or gadolinium-enhancing lesions on brain MRIs.

The primary endpoints were the presence of relapses and the time to first relapse, which were assessed for both groups using surveillance analysis from two different starting points: STRm (which includes the washout period for the JCV + OCR arm) and time of OCR initiation (which excludes the washout period for the JCV + OCR arm). The secondary endpoints were the annualized relapse rate (ARR) one year after STRm and after OCR initiation, and the increase in EDSS score, presence of gadolinium-enhancing or new T2-hyperintense lesions, and the proportion of NEDA one year after STRm.

In the multivariate analysis, variables included were sex, age at treatment initiation, treatment (NTZ or OCR), ARR 2 years before STRm, EDSS at STRm, and time on NTZ before STRm.

### Statistical analysis

Primary endpoints were assessed using Kaplan–Meier survival analysis and the log-rank test. Secondary endpoints were assessed using bivariate and multivariate analyses as appropriate. Categorical variables are presented as absolute frequencies. Demographic and clinical variables are presented as medians, ranges, means, and standard deviations according to the distribution. Binomial negative regression test, chi-square test and Fisher exact test were used as bivariate tests, as appropriate, and Cox regression by forward stepwise selection was used as a multivariate test. All tests were conducted at 95% confidence intervals and at a significance level of 5%. Statistical analyses were performed using SPSS version 20 (SPSS Inc., Chicago, IL, USA).

### Standard protocol approvals, registrations, and patient consents

This study was approved by the Hospital Universitari de Bellvitge Research Ethics Committee, with reference EOM031/22. Patients signed informed consent forms, and data were collected anonymously. Patient information confidentiality was handled in accordance with Spanish regulations.

### Data availability

The datasets used and/or analyzed during this study are available from the corresponding author upon reasonable request from any qualified investigator. The corresponding author takes full responsibility for the data, the analyses and interpretation, and the conduct of the research. The corresponding author has full access to all of the data and the right to publish any and all data separate and apart from any sponsor.

## Results

**Fig. 1:**
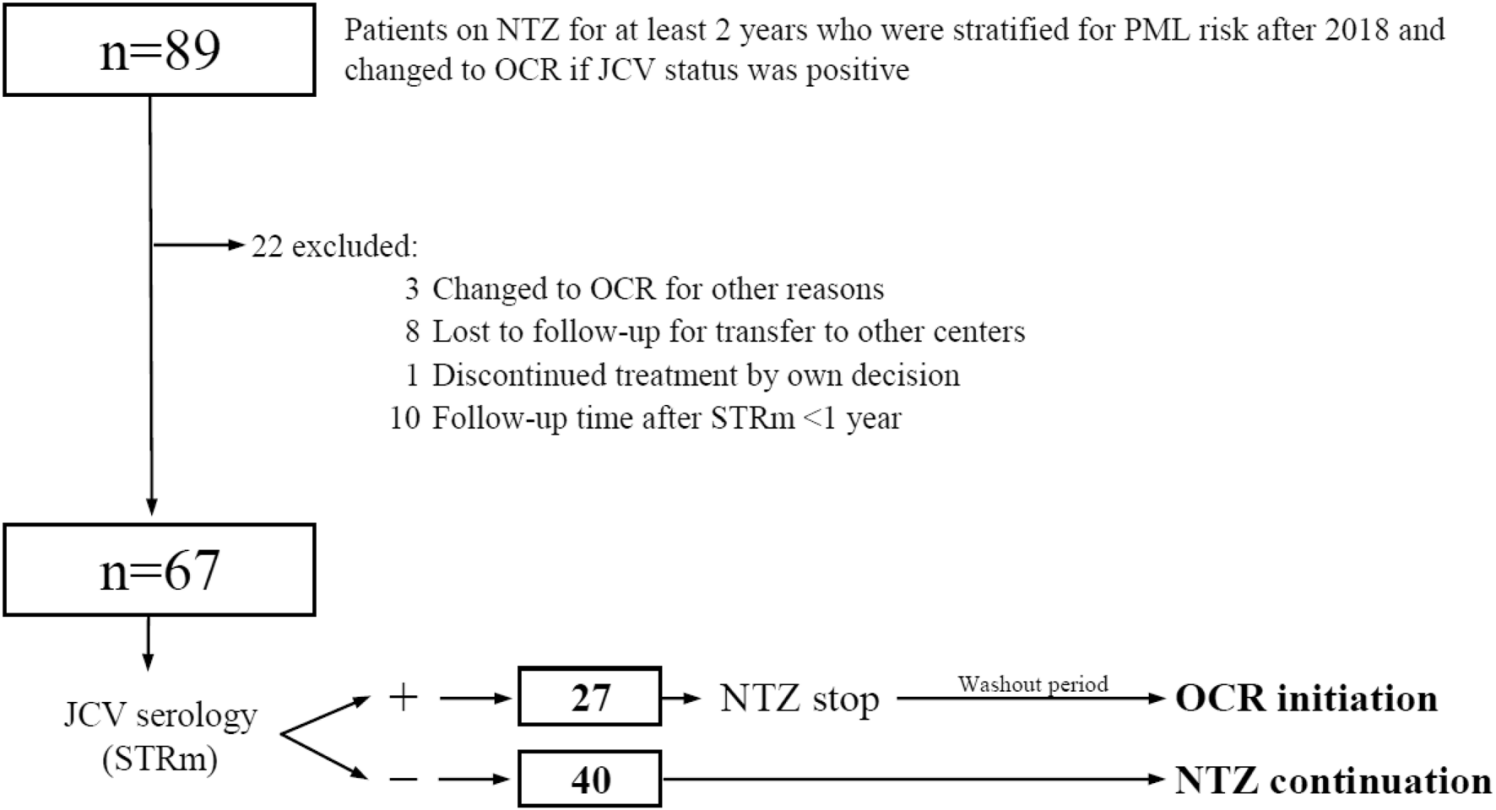
Flowchart for patient inclusion

Of 96 patients screened, 7 were initially excluded for switching to a treatment other than OCR after JCV seroconversion (5 to fingolimod and 2 to rituximab). Of the remaining 89 patients, 67 were included in the study. Of these, 40 continued treatment with NTZ (60%), and 27 changed to OCR (40%) (Fig. 1).

All patients in the JCV + OCR arm started the new therapy after 2019. The median washout time from NTZ discontinuation to the first OCR infusion was 94 days (IQR 85–101). The demographic and clinical baseline data are shown in Table 1. The median follow-up time was 2.99 (IQR 1.77–3.44) for the JCV-NTZ arm and 2.44 (IQR 2.31–2.72) for the JCV + OCR arm.

**Table 1:** Baseline characteristics of each group

	JCV-NTZ (*n* = 40)	JCV + OCR (*n* = 27)	*p* value
Age at MS onset, years (mean ± SD)	29.53 ± 8.25	27.24 ± 9.06	0.298
Age at STRm, years (mean ± SD)	41.35 ± 10.01	44.99 ± 9.61	0.332
Female, *n* (%)	30 (75.0%)	19 (70.4%)	0.675
Number of DMTs before NTZ, *n* (mean ± SD)	1.08 ± 0.99	1.22 ± 0.58	0.491
Annualized relapse rate before NTZ, mean ± SD	1.40 ± 0.78	1.48 ± 0.80	0.679
Time with NTZ before STRm, years (mean ± SD)	3.56 ± 1.96	6.15 ± 3.51	0.001
Annualized relapse rate two years before STRm, mean ± SD	0.13 ± 0.27	0.20 ± 0.32	0.282
EDSS score at STRm, mean ± SD	2.79 ± 1.58	3.37 ± 1.50	0.121
Gd + lesions on last IRM before STRm, *n*	0	0	

**Fig. 2:**
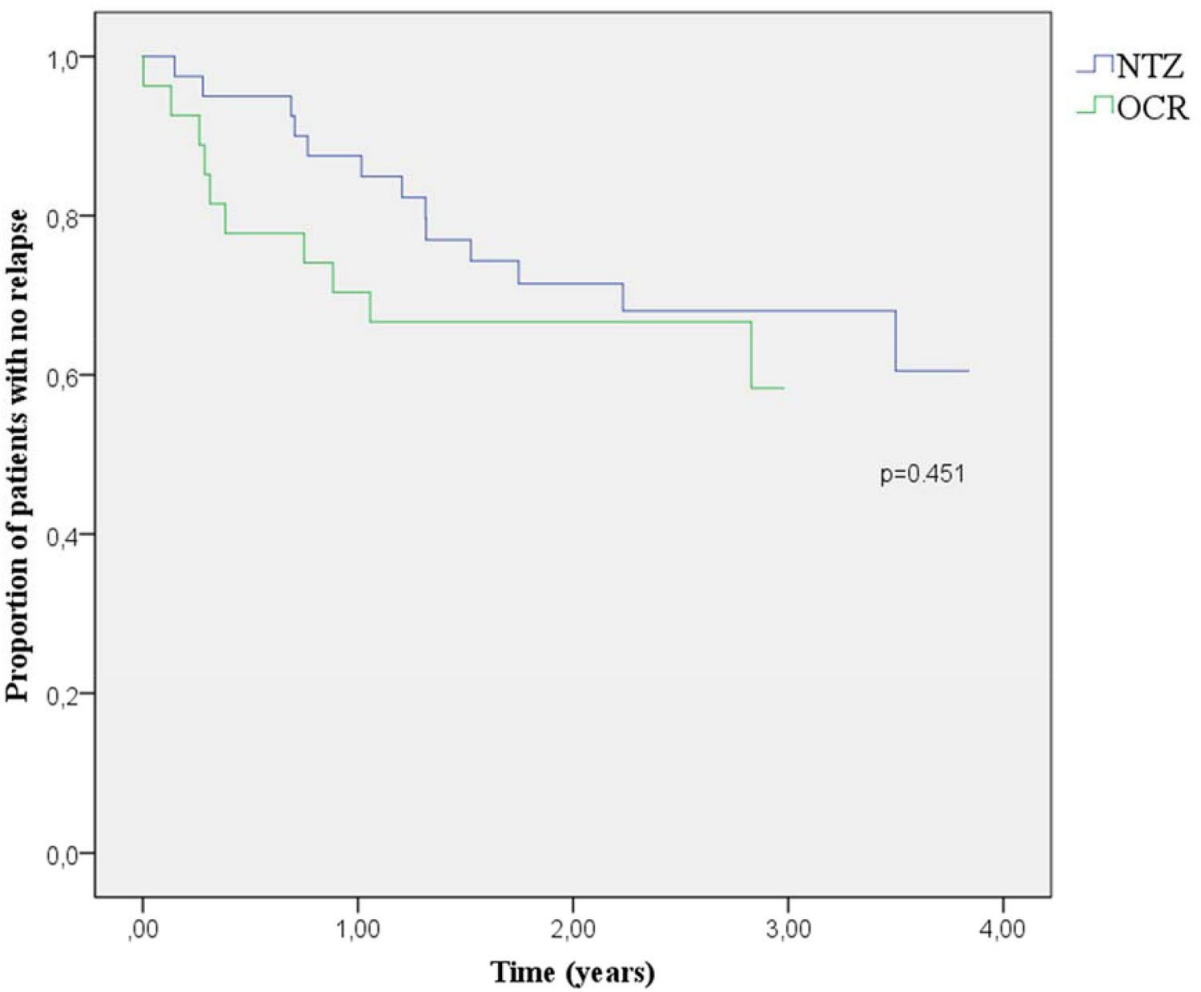
Kaplan–Meier surveillance since STRm for both arms of treatment

**Fig. 3:**
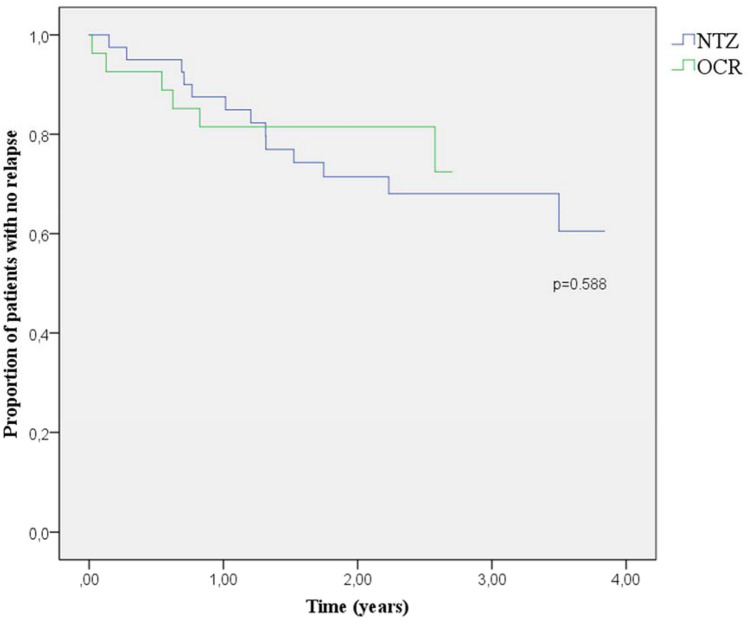
Kaplan–Meier surveillance since OCR initiation (for the JCV + OCR arm) or STRm (for the JCV-NTZ arm)

Time to first relapse from the STRm and OCR initiation was not significantly different between groups (0.75 ± 0.42 years since STRm and 2.58 years since OCR initiation for the JCV + OCR arm, versus 1.53 ± 0.51 years for the JCV-NTZ arm; p75, *p* = 0.451 and 0.588 respectively) (Figs. 2 and 3). Ten patients in the JCV + OCR arm presented a relapse after STRm (37%), four during the washout period and before the first dose of treatment, and 13 patients in the JCV-NTZ arm (32.5%). Though not statistically significant, the ARR in the first year after STRm was lower for the JCV-NTZ arm (0.18 for NTZ versus 0.33 for OCR, chi-square value = 1.64, *p* = 0.201), and this difference appears lower when the washout period is excluded and the ARR is assessed after OCR initiation (0.22 for OCR, *p* = 0.691). No differences in the increase in EDSS score, new MRI lesions, and proportion of NEDA were detected between the groups. The endpoint characteristics are summarized in Table 2.

**Table 2:** Endpoint variables for each group

	JCV-NTZ (*n* = 40)	JCV + OCR (*n* = 27)	*p* value
Presence of relapse during the follow-up, *n* (%)			
After STRm	13 (32.5%)	10 (37.0%)	0.701
After OCR initiation	13 (32.5%)	6 (22.2%)	0.360
Time to first relapse, years (p75 ± SE)			
After STRm	1.525 ± 0.507	0.750 ± 0.424	0.451
After OCR initiation	1.525 ± 0.507	2.576	0.588
Annualized relapse rate in the first year, mean ± SD			
After STRm	0.18 ± 0.45	0.33 ± 0.55	0.201
After OCR initiation	0.18 ± 0.45	0.22 ± 0.51	0.691
Increase in EDSS score after 1 year, n (%)	1 (2.6%)	0	0.402
New T2-hyperintense lesions after 1 year, *n* (%)	0	1 (3.7%)	0.22
Gd-enhancing lesions after 1 year, *n* (%)	0	0	
NEDA 1 year after STRm, *n* (%)	33 (82.5%)	19 (70.4%)	0.243
NEDA 2 years after STRm, *n* (%)	23 (65.7%)	17 (63.0%)	0.516

In multivariate analysis, no statistically significant association was found between the time to first relapse and the previously mentioned variables.

## Discussion

This study was based on a natural experiment with patients assigned to each treatment group, depending on the JCV status. As JCV status has not been related to multiple sclerosis prognosis (excluding the occurrence of PML), it works as a natural randomization factor. This pseudo-randomization is not controlled by any investigator and is not affected by the patient’s characteristics, depending only on a natural phenomenon, thus allowing the comparison of groups with low selection bias. To our knowledge, this is the first time that JCV would be used as a random natural randomization factor, though other natural experiments have previously been used in health research, such as in mendelian randomization [21].

No significant differences in the time to first relapse were observed between NTZ continuation and OCR treatment in our cohort. Although not statistically significant, earlier relapses in the JCV + OCR group are visible in Fig. 2, especially during the washout period, leading to further relapse stabilization after the first year. In fact, the annualized relapse rate in the first year after STRm seems higher in the JCV + OCR group, though not statistically significant, and is reduced by evaluating it since OCR initiation and excluding the washout period. No differences were found in NEDA status, progression, or radiological activity after 1 year. This finding is probably explained by the rebound effect of NTZ discontinuation, as previously reported [13, 14].

Nonetheless, on comparing both groups since OCR real initiation (and not since the STRm when positive JCV was detected), no statistical differences were detected in first-year relapse activity (Fig. 3). This beneficial difference in the time to first relapse from the previous comparison shows the effect of relapse occurring during the washout period. All washout periods were at least 60 days, in accordance with the current institutional guidelines of our health department. With these timings, the risk of rebound effect after NTZ withdrawal increases, but it probably minimizes the risk of carryover PML [10], which was strictly screened with MRI within three months prior to OCR initiation and after three months, and was never found in our cohort.

When comparing treatments since OCR initiation, no differences in disease activity were observed between NTZ and OCR, as previously suggested by other authors [22]. In fact, a non-statisticallymsignificant lower presence of further relapse and longer time to first relapse was observed for OCR, suggesting a potential superiority compared to NTZ. Special consideration must be taken from this affirmation, as the effect of OCR is probably delayed for a few months after initiation, and the follow-up time for the OCR arm is shorter. Moreover, this sample of patients was under an incontrollable selection bias, as they were all NTZ responders who had been on NTZ for more than two years.

A relatively high proportion of relapses was observed for both treatment arms (32.5% for JCV-NTZ and 37.0% for JCV + OCR), though this includes the totality of the follow-up, with a median time of almost 3 years for the JCV-NTZ arm and 2.5 years for the JCV + OCR arm, therefore it is reduced when analyzing the ARR only in the first year (0.18 ± 0.45 vs 0.33 ± 0.55, respectively). Furthermore, no change in EDSS or MRI was required for the definition of relapse, potentially allowing a broader inclusion of relapses that should not differ between groups.

It must be considered that the time on NTZ pre-STRm was higher for the JCV + OCR group, although this is not surprising, as a longer exposure to NTZ would have implied a major number of JCV testing and so a higher cumulative probability of JCV positivity [23]. In addition, the JCV conversion rate has been found to be higher in NTZ-treated patients than in healthy controls, supporting a possible effect of NTZ on the immune system that may be trophic for JCV seroconversion [24]. However, as revealed in the multivariate analysis, this difference in time for NTZ pre-STRm does not affect the outcomes of our cohort.

Although our model is free of selection bias, some limitations of the present study must be considered. First, our sample had a limited number of patients and a low incidence of events during follow-up. This glaring lack of sample size may have led to the non-significant differences in the results, which probably does not reflect what is clinically significant, as for instance, time to first relapse was twice as long for the JCV-NTZ arm (1.53 years) compared to the JCV + OCR arm (0.75 years). Second, though data were collected in a prospective manner, the analysis and study design were retrospective, leading to possible information biases or confounding factors.

In this study, we investigated a cohort of patients who were switched to OCR. However, it is important to note that other treatment alternatives can be considered when JCV turns positive after two years of NTZ. Fingolimod offers a viable alternative, with the comfort of oral administration; however, its adverse effects are quite common (including the risk of PML and rebound effect) [25], and its efficacy has been proven to be lower compared to other high-efficacy treatments, such as NTZ itself [26–29]. Cladribine offers the chance of an easy posology, which is compatible with family planning, and has shown superior efficacy compared to other oral treatments [30, 31]. Lastly, alemtuzumab induction therapy can be of interest in certain patients with high activity after the discontinuation of NTZ [32, 33] but with a notable risk of serious adverse events, including secondary autoimmunity, which requires considerable monitoring [34, 35].

In multiple sclerosis, the comparison of highly effective disease-modifying treatments is an unsolved challenge. Randomized, double-blind clinical trials for treatment comparison are missing, probably due to ethical and economic reasons, but also due to the low incidence of events in these treated patients, which would require the design of trials with very long follow-ups. Therefore, conclusions from treatment comparisons must be based on real-world evidence from observational studies. In this context, designing studies based on natural experiments provides an opportunity for pseudo-randomization, minimizing the selection biases that are inherent to observational designs.

## Conclusion

In conclusion, the JCV status can be used as a natural experiment to compare treatment arms with low selection bias. In our study, switching to OCR versus NTZ continuation led to similar disease activity outcomes but with a possible increase in relapses in the first year of OCR switching, likely related to a prolonged washout period.
